# Author Correction: Stochastic simulations reveal few green wave surfing populations among spring migrating herbivorous waterfowl

**DOI:** 10.1038/s41467-019-11151-7

**Published:** 2019-07-22

**Authors:** Xin Wang, Lei Cao, Anthony D. Fox, Richard Fuller, Larry Griffin, Carl Mitchell, Yunlin Zhao, Oun-Kyong Moon, David Cabot, Zhenggang Xu, Nyambayar Batbayar, Andrea Kölzsch, Henk P. van der Jeugd, Jesper Madsen, Liding Chen, Ran Nathan

**Affiliations:** 1 State Key Laboratory of Urban and Regional Ecology, Research Center for Eco-Environmental Sciences, Chinese Academy of Sciences, 100085 Beijing, China; 2 Movement Ecology Laboratory, Department of Ecology, Evolution and Behavior, Alexander Silberman Institute of Life Sciences, The Hebrew University of Jerusalem, 91904 Jerusalem, Israel; 3 University of Chinese Academy of Sciences, 100049 Beijing, China; 4 Department of Bioscience, Aarhus University, Kalø, Grenåvej 14, DK-8410 Rønde, Denmark; 5 School of Biological Sciences, University of Queensland, Brisbane, QLD 4072 Australia; 6 The Wildfowl & Wetlands Trust (WWT), Slimbridge, Gloucestershire GL2 7BT UK; 7 College of Life Science and Technology, Central South University of Forestry and Technology, 410004 Changsha, China; 8 Animal and Plant Quarantine Agency, Gimcheon, 39660 Republic of Korea; 9 School of Biological, Earth and Environmental Science, University College Cork, Distillery Fields, North Mall, Cork, T23 N73K Ireland; 10 Wildlife Science and Conservation Center, B-802 Union Building, Sukhbaatar District, Ulaanbaatar, 14210 Mongolia; 11 Department of Migration and Immuno-Ecology, Max Planck Institute for Ornithology, Am Obstberg 1, 78315 Radolfzell, Germany; 12 Group of Mathematical Modelling, Institute for Chemistry and Biology of the Marine Environment, Carl von Ossietzky University Oldenburg, Carl-von-Ossietzky-Straße 9-11, 26111 Oldenburg, Germany; 13 Institute for Wetlands and Waterbird Research e.V. (IWWR), Am Steigbügel 3, 27283 Verden(Aller), Germany; 14 Vogeltrekstation—Dutch Centre for Avian Migration and Demography (NIOO-KNAW), Wageningen, 6708 PB The Netherlands; 15 Sovon Dutch Centre for Field Ornithology, PO Box 65216503, GA Nijmegen, The Netherlands

**Keywords:** Animal migration, Animal migration

Correction to: *Nature Communications* 10.1038/s41467-019-09971-8, published online 16 May 2019.

The original version of this Article incorrectly listed the affiliation of ‘Zhenggang Xu’ as ‘The Wildfowl & Wetlands Trust (WWT), Slimbridge, Gloucestershire GL2 7BT, UK’, instead of the correct ‘College of Life Science and Technology, Central South University of Forestry and Technology, 410004 Changsha, China’. This has been corrected in both the PDF and HTML versions of the Article.

