# Supplementary material for: Stochastic simulations reveal few green wave surfing populations among spring migrating herbivorous waterfowl
Source: Nat Commun. 2019 May 16;10:2187. doi: 10.1038/s41467-019-09971-8 (PMC6522631; doi:10.1038/s41467-019-09971-8)
Supplement: Supplementary file 1 — Supplementary Information [file 41467_2019_9971_MOESM1_ESM.docx]

Stochastic simulations reveal few green wave surfing populations among spring migrating herbivorous waterfowl

Wang et al.

**Supplementary Information**

**Contents**

[Supplementary Table 1 2](#_Toc1632710)

[Supplementary Table 2 3](#_Toc1632711)

[Supplementary Table 3 4](#_Toc1632712)

[Supplementary Table 4 8](#_Toc1632713)

[Supplementary Table 5 10](#_Toc1632714)

[Supplementary Table 6 13](#_Toc1632715)

[Supplementary Table 7 15](#_Toc1632716)

[Supplementary Figure 1 16](#_Toc1632717)

[Supplementary Figure 2 19](#_Toc1632718)

[Supplementary Figure 3 20](#_Toc1632719)

[Supplementary Figure 4 21](#_Toc1632720)

[Supplementary Figure 5 22](#_Toc1632721)

[Supplementary Figure 6 23](#_Toc1632722)

[Supplementary Figure 7 24](#_Toc1632723)

[Supplementary Figure 8 26](#_Toc1632724)

[Supplementary Figure 9 27](#_Toc1632725)

[Supplementary Figure 10 28](#_Toc1632726)

[Supplementary Figure 11 29](#_Toc1632727)

[Supplementary References 30](#_Toc1632728)

Supplementary Table 1**. Summary of Simple Conventional Correlation, Correlation method evaluated by Stochastic Migrations and Metric Selection approach based on Stochastic Migration tests of migration-green wave associations.**

|  | Simple Conventional Correlation | Correlation method evaluated by Stochastic Migrations | Metric Selection approach based on Stochastic Migration tests of migration-green wave associations |
| --- | --- | --- | --- |
| Response variable | Observed arrival day at stopover sites | Pearson correlation coefficient of observed migrations of green wave surfers/weak surfers identified by Simple Conventional Correlation | Green wave metrics for observed stopover |
| Explanatory variable | Predicted arrival day with peak green-up rate | Pearson correlation coefficient of three types of stochastic migrations | Green wave metrics for simulated stopover in the three types of stochastic migrations |
| Evaluation methods | Examining the slope of linear mixed-effects models | Examining the difference of correlation coefficients between observed and stochastic migrations | Multiple comparisons |
| Results and interpretation | Slope=1 and intercept=0: green wave surfer; Slope>1 or 0<slope<1 regardless of intercept, or slope>0 with intercept≠0: weak surfer; Otherwise non-surfer. | The correlation coefficient of observed migration of green wave surfers (identified by Simple Conventional Correlation) is significantly higher than all stochastic migrations: surfer; Otherwise non-surfer. | Observed green wave metrics significantly different from all stochastic ones: surfer; otherwise non-surfer. |

Supplementary Table 2**. Comparison of three types of stochastic migration simulations.**

|  | Stochastic timing | Stochastic stopover site | Stochastic timing and stopover site |
| --- | --- | --- | --- |
| **Temporal elements** | | | |
| (1) Starting time of migration, (2) non-stopover period during migration, and (3) number of stopover sites | Same as the observed migration | Same as the observed migration | Same as the observed migration |
| (4) Stopover duration at each site | Randomly drawn from the observed stopover duration pool of the corresponding population, controlling for the latest arrival at breeding site | Same sequence and duration as the observed migration | Randomly drawn from the observed stopover duration pool of the corresponding population, controlling for the latest arrival at breeding site |
| **Spatial elements** | | | |
| (1) Migration track | Same as the observed migration | Stochastic tracks based on same parameters of continuous-time correlation random walk models | |
| (2) Location of stopover site | Same as the observed migration | Randomly assigned from stochastic migration tracks, controlling for the minimum distance between stopover sites and maximum distance of migration flight. | |

Supplementary Table 3**. Overview of the spring migration dataset, including source, sample size, geographical range and feeding guilds of the study species.**

| Data type | Data source | Location data type | Species | Geographical range | Duration | Abbreviation | Feeding guild | No. birds | No. bird ∙year | No. stopover sites | Mean±SD stopover duration (days) | Mean±SD maximum step length within stopover sites (km)^*^ | Coefficient of variance of stopover duration | Total no. of locations | Mean±SD no. of daily locations of bird-year | Mean±SD of tracking period of bird-year (days) | Range (min,max) of tracking period of bird-year (days) | Mean±SD of tracking distance of bird-year (km) |
| --- | --- | --- | --- | --- | --- | --- | --- | --- | --- | --- | --- | --- | --- | --- | --- | --- | --- | --- |
| Published tracks | Cabot^1^, Kölzsch *et al.*^2^ | GPS | Barnacle Goose *Branta leucopsis* | Western Europe - Greenland | 2008-2010 | BGoo_Gre | Grazer^3^ | 7 | 7 | 21 | 9.29±6.27 | 12.31±9.40 | 0.68 | 2734 | 10.62±3.35 | 35.88±3.06 | (31.2,38.71) | 2782.19±512.39 |
| Published tracks | Griffin^4^, Kölzsch *et al.*^2^ | GPS | Barnacle Goose *B. leucopsis* | Western Europe - Svalbard | 2006-2011 | BGoo_Sva | Grazer^3^ | 13 | 15 | 24 | 11.38±9.6 | 6.65±9.29 | 0.85 | 3889 | 8.43±1.63 | 31.01±15.77 | (14.41,71) | 3090.33±207.69 |
| Published tracks | Van Der Jeugd *et al.*^5^, Kölzsch *et al.*^2^ | GPS | Barnacle Goose *B. leucopsis* | Western Europe - Barents Sea | 2008-2011 | BGoo_Bar | Grazer^3^ | 13 | 27 | 93 | 13.66±15.22 | 9.47±7.83 | 1.12 | 6276 | 3.76±0.23 | 60.71±25.29 | (18.7,100.91) | 3326.99±421.64 |
| Published tracks | Kölzsch *et al.*, Kölzsch *et al.*^6^ | GPS/GPS+Argos^†^ | Greater White-fronted Goose *Anser albifrons* | Western Europe - Barents Sea | 2006-2014 | GWGoo_Bar | Grazer^7^ | 45 | 45 | 262 | 9.79±8.57 | 19.35±19.30 | 0.88 | 46929 | 14.99±18.68 | 70.82±21.24 | (13.81,125.3) | 4658.65±1393.90 |
| Migration tracks by this study |  | GPS | Greater White-fronted Goose *A. albifrons* | Eastern China - Eastern Russia | 2015-2016 | GWGoo_EA | Grazer^8^ | 30 | 31 | 120 | 12.17±11.37 | 24.63±22.21 | 0.94 | 32093 | 17.08±6.23 | 56.13±26.90 | (2.4,126.7) | 5010.48±1757.38 |
| Stopover sites in literature | Kanai *et al.*^9^ | GPS | Whooper Swan *Cygnus cygnus* | Japan - Eastern Russia | 1994-1995 | WSwan_EA | Facultative herbivore^10^ | 5 | 5 | 19 | 14.48±10.08 |  | 0.7 |  |  |  |  |  |
| Migration tracks by this study |  | GPS | Tundra Swan *C. columbianus* | Eastern China - Eastern Russia, North Europe | 2014-2016 | TSwan_EA | Facultative herbivore^11^ | 13 | 13 | 29 | 9.49±6.93 | 9.05±9.13 | 0.74 | 6015 | 17.41±7.33 | 26.98±18.11 | (5.41,62.3) | 2052.64±710.13 |
| Migration tracks by this study |  | GPS | Swan Goose *A. cygnoides* | Eastern China - Eastern Russia | 2015-2016 | SGoo_EA | Facultative herbivore^12^ | 9 | 9 | 28 | 11.08±10.16 | 14.61±14.83 | 0.92 | 3250 | 9.29±6.44 | 37.59±18.35 | (12.31,72) | 2880.45±754.59 |
| Migration tracks by this study |  | GPS | Taiga Bean Goose *A. fabalis* | Western Europe - Central Scandinavia | 2013-2016 | BnGoo_Sca | Facultative herbivore^13^ | 7 | 7 | 14 | 14.36±8.83 | 12.03±15.50 | 0.62 | 7095 | 18.15±13.83 | 53.76±10.23 | (41.11,61.91) | 2069.25±295.88 |
| Migration tracks by this study |  | GPS | Tundra Bean Goose *A. serrirostris* | Eastern China - Eastern Russia | 2014-2016 | BnGoo_EA | Facultative herbivore^14^ | 10 | 10 | 36 | 12.45±8.70 | 28.06±25.71 | 0.7 | 3986 | 7.87±6.24 | 53.41±24.05 | (8.31,91.5) | 3948.51±1762.67 |
| Published tracks | Chudzińska *et al.*^15^, Chudzińska and Madsen^16^ | GPS | Pink-footed Goose *A. brachyrhynchus* | Western Europe - Svalbard | 2011-2012 | PGoo_Sva | Facultative herbivore^15^ | 8 | 10 | 25 | 12.45±8.51 | 32.90±20.78 | 0.69 | 3485 | 13.67±1.30 | 49.77±14.01 | (31.4,78.1) | 2775.39±493.77 |
| Migration tracks by this study |  | GPS/GPS+Argos | Greater White-fronted Goose *A. albifrons* | Western Europe - Greenland | 2008, 2013-2016 | GWGoo_Gre | Facultative herbivore^17^ | 18 | 28 | 36 | 17.84±8.55 | 9.88±8.54 | 0.48 | 7436 | 8.96±3.58 | 28.48±4.70 | (20.2,40.21) | 3230.66±335.43 |
| Migration tracks by this study |  | GPS | Mallard *Anas platyrhynchos* | Eastern China - Northeastern China | 2016 | Mal_EA | Omnivores^18^ | 7 | 7 | 21 | 12.86±10.98 | 10.88±10.94 | 0.86 | 1806 | 5.83±0.09 | 42.99±17.76 | (18.2,68.41) | 1644.31±506.66 |
| Stopover sites in literature | Miller *et al.*^19^ | GPS | Northern Pintail *Anas acuta* | North America | 2001 | NP_NA | Omnivores^10^ | 8 | 8 | 31 | 23.62±16.99 |  | 0.72 |  |  |  |  |  |

Data from a total of 193 birds (222 migration episodes) comprising a total of 124,994 GPS locations were retained for use in the correlation analysis and to generate stochastic simulations (n = 1,000 each scheme, see Supplementary Table 1, Supplementary Fig. 8). See Supplementary Data 1 for more details of species, tracking period and loggers. ^*^ Mean maximum step length within stopover sites is the population-level mean of the maximum distance between any pair of locations within each stopover site. ^†^ GPS/GPS+Argos, and hereafter, means that the study used both GPS loggers and GPS+Argos loggers, which provided both GPS and Argos locations. However, we only included GPS locations in all analyses in this study and in this overview table, because (1) Argos locations involved much higher error than GPS locations, (2) the GPS+Argos loggers provided far fewer Argos locations than GPS locations, and (3) the simultaneous working of the two location systems will result in duplicated timestamped locations.

Supplementary Table 4**. AICc-based model selection of the mixed effect models explaining the variation in the Simple Conventional Correlation, Correlation method evaluated by Stochastic Migrations and Metric Selection based on Stochastic Migrations (MSSM) modelling by bill morphology using three categorizing schemes of response variable.**

| Models | Df | logLik | AICc | ΔAICc | Marginal R^2^ | Conditional R^2^ |
| --- | --- | --- | --- | --- | --- | --- |
| **Simple Conventional Correlation - Scheme 1^*^** |  |  |  |  |  |  |
| Intecept | 2 | -3.60 | 12.3 | 0 | 0.000 | 0.000 |
| Intercept-52.99×dlRatio | 3 | -2.14 | 12.7 | 0.38 |  |  |
| Intercept+BioGrp | 4 | -1.39 | 15.2 | 2.92 |  |  |
| Intercept-1518×dlRatio+BioGrp | 5 | 0 | 17.5 | 5.20 |  |  |
| **Simple Conventional Correlation - Scheme 2** |  |  |  |  |  |  |
| Intecept | 3 | -3.62 | 15.6 | 0 | 0.000 | 0.000 |
| Intercept-1.08×dlRatio | 4 | -3.61 | 19.7 | 4.02 |  |  |
| Intercept+BioGrp | 5 | -2.70 | 22.9 | 7.27 |  |  |
| Intercept+1.23×dlRatio+BioGrp | 6 | -2.48 | 29.0 | 13.32 |  |  |
| **Simple Conventional Correlation - Scheme 3** |  |  |  |  |  |  |
| Intecept | 2 | -9.18 | 23.4 | 0 | 0.000 | 0.343 |
| Intercept+1.38×dlRatio | 3 | -9.17 | 26.7 | 3.29 |  |  |
| Intercept+BioGrp | 4 | -8.53 | 29.5 | 6.05 |  |  |
| Intercept+18.02×dlRatio+BioGrp | 5 | -8.04 | 33.6 | 10.14 |  |  |
| **Correlation method evaluated by Stochastic Migrations**^†^ |  |  |  |  |  |  |
| Intecept | 2 | -8.85 | 22.8 | 0 | 0.000 | 0.264 |
| Intercept+11.94×dlRatio | 3 | -8.08 | 24.6 | 1.78 |  |  |
| Intercept+BioGrp | 4 | -7.56 | 27.6 | 4.78 |  |  |
| Intercept+20.02×dlRatio+BioGrp | 5 | -6.87 | 31.2 | 0.46 |  |  |
| **MSSM (using the metric IRG)**^†^ |  |  |  |  |  |  |
| Intercept+140.9×dlRatio | 3 | -3.18 | 14.8 | 0 | 0.698 | 0.980 |
| Intecept | 2 | -6.19 | 17.5 | 2.71 |  |  |
| Intercept+BioGrp | 4 | -5.67 | 23.8 | 9.02 |  |  |
| Intercept+140.9×dlRatio+BioGrp | 5 | -3.18 | 23.9 | 9.1 |  |  |

The explanatory variables include biological group (BioGrp) and upper mandible depth-length ratio (dlRatio). Marginal/conditional R^2^ represents the variance explained by fixed effects/both fixed and random effects^20,21^. Scheme 1: mixed effect logistic model, considering only surfer as 1 and others 0; Scheme 2: mixed linear model, considering surfer as 1, weak surfer 0.5, and non-surfer 0; Scheme 3: mixed effect logistic model, considering surfer and weak surfer as 1 and non-surfer 0. ^*^The candidate model that contained only upper mandible depth-length ratio was excluded because of convergence failure. ^†^Mixed effect logistic model, considering surfer as 1 and others 0.

Supplementary Table 5**. Statistics of Simple Conventional Correlation of arrival time, and Correlation method evaluated by Stochastic Migrations for migratory grazing, facultative herbivorous and omnivorous Anatidae.**

| Population abbreviation^*^ | Simple Conventional Correlation | | | | | |  | Variance components of the mixed linear models of Simple Conventional Correlation | | | | | Simple-correlation-classified surfing type | Correlation method evaluated by Stochastic Migrations - the Pearson correlation coefficient / 95% CI (lower, upper) of observed and stochastic migrations | | | | |
| --- | --- | --- | --- | --- | --- | --- | --- | --- | --- | --- | --- | --- | --- | --- | --- | --- | --- | --- |
|  | Coefficient of Day of 50% GWI (slope)^*^ | 95%CI of slope (lower, upper) | P value of slope | 95%CI of intercept (lower, upper) | P value of intercept | 95% quantile interval of the difference between observed and green-wave-expected arrival timing in days^†^ (lower, upper) |  | Slope | | Intercept | | Residual |  | Observed migration | Stochastic timing | Stochastic stopover site | Stochastic timing and stopover sites |  |
|  |  |  |  |  |  |  |  | Year^‡^ | Individual bird nested in year^§^ | Year^‡^ | Individual bird nested in year^§^ |  |  |  |  |  |  |  |
| BGoo_Gre | 0.34 | (-0.16,0.83) | 0.16 | (-2.05,144.25) | 0.06 | (-56.09,38.20) |  |  | 0.26 |  | 5664.96 | 4.64 | None | ^‖^ | ^‖^ | ^‖^ | ^‖^ |  |
| BGoo_Sva | 0.33 | (0.04,0.63) | 0.04 | (36.40,118.92) | 0.00 | (-46.37,23.67) |  | 0.01 | 0.01 | 335.37 | 1.15 | 126.38 | **Weak surfer** | 0.69 | (0.19,0.22) | (0.28,0.31) | (0.19,0.22) |  |
| BGoo_Bar | 0.55 | (0.36,0.74) | 0.01 | (28.20,81.40) | 0.00 | (-48.77,48.34) |  | 0.01 | 0.07 | 194.56 | 1470.03 | 259.84 | **Weak surfer** | 0.67 | (0.34,0.35) | (0.42,0.44) | (0.40,0.42) |  |
| GWGoo_Bar | 0.71 | (0.64,0.79) | <0.001 | (11.82,29.68) | <0.001 | (-63.99,14.41) |  |  | 0.01 |  | 24.18 | 222.17 | **Weak surfer** | 0.81 | (0.57,0.58) | (0.60,0.61) | (0.53,0.54) |  |
| GWGoo_EA | -0.17 | (-0.53,0.18) | 0.33 | (84.40,206.06) | <0.001 | (-140.61,1.18) |  | 0.05 | 0.04 | 1108.78 | 1544.09 | 413.54 | None | ^‖^ | ^‖^ | ^‖^ | ^‖^ |  |
| WSwan_EA | 0.95 | (0.58,1.32) | 0.01 | (-79.16,34.60) | 0.41 | (-63.21,-7.50) |  |  | 0.01 |  | 520.05 | 130.68 | **Surfer** | 0.74 | (0.52,0.57) | ^¶^ | ^¶^ |  |
| TSwan_EA | -0.06 | (-0.24,0.12) | 0.47 | (74.42,134.61) | <0.001 | (-138.82,-3.35) |  |  | 0.01 |  | 12.31 | 209.19 | None | ^‖^ | ^‖^ | ^‖^ | ^‖^ |  |
| SGoo_EA | -0.18 | (-0.35,-0.00) | 0.05 | (96.31,154.68) | <0.001 | (-133.03,2.86) |  |  | 0.01 |  | 1.38 | 180.91 | None | ^‖^ | ^‖^ | ^‖^ | ^‖^ |  |
| BnGoo_Sca | -0.65 | (-1.59,0.29) | 0.15 | (39.93,291.73) | 0.02 | (-69.89,-11.37) |  |  | 0.4 |  | 7352.8 | 103.02 | None | ^‖^ | ^‖^ | ^‖^ | ^‖^ |  |
| BnGoo_EA | -0.11 | (-0.37,0.15) | 0.38 | (62.53,154.64) | <0.001 | (-160.65,-0.29) |  |  | 0.08 |  | 2398.78 | 270.47 | None | ^‖^ | ^‖^ | ^‖^ | ^‖^ |  |
| PGoo_Sva | 0.41 | (0.15,0.67) | 0.01 | (22.76,89.31) | 0.00 | (-55.65,0.79) |  | 0.01 | 0.06 | 0.06 | 806.39 | 79.94 | **Weak surfer** | 0.66 | (0.35,0.37) | (0.28,0.32) | (0.26,0.30) |  |
| GWGoo_Gre | -0.02 | (-0.20,0.16) | 0.79 | (80.12,139.81) | <0.001 | (-101.28,-5.83) |  | 0.01 | 0.01 | 429.8 | 0.06 | 58.17 | None | ^‖^ | ^‖^ | ^‖^ | ^‖^ |  |
| Mal_EA | -0.06 | (-0.39,0.26) | 0.65 | (87.53,184.30) | <0.001 | (-134.12,-3.05) |  |  | 0.1 |  | 1959.2 | 72.35 | None | ^‖^ | ^‖^ | ^‖^ | ^‖^ |  |
| NP_NA | 0.72 | (0.34,1.10) | 0.01 | (4.45,74.28) | 0.03 | (-68.12,71.47) |  |  | 0.06 |  | 20.09 | 507.85 | **Weak surfer** | 0.27 | (0.48,0.51) | ^¶^ | ^¶^ |  |

Both methods fit a linear regression model for the observed arrival day of birds to a stopover site along the spring migration track versus the predicted arrival day defined as the day with 50% GWI. Migratory populations are classified as green wave surfers, weak surfers or non-surfers if the slope of this regression (the 50% GWI) is, respectively, not statistically different from 1, lower than 1 and higher than 0, and lower than or not statistically different from 0. The Correlation method evaluated by Stochastic Migrations simulates stochastic timing, stochastic stopover site, stochastic timing and stopover site spring (northward) migrations. Using 1,000 simulations of each type, we estimated the probability of obtaining the simple-correlation-classified surfing type (or a better fit to the green wave hypothesis) by stochastic northward migration irrespective of the green wave. High probability implies that the simple-correlation-classified surfing type is likely to be attributed to proximate driver(s) other than the green wave. Note that the in most cases (12/14) the 95% CI values of the difference between the observed migration timing and the green wave timing are negative, implying that birds generally arrived to stopover sites earlier than the green wave. ^*^Abbreviations are defined in Supplementary Table 3; ^†^Deviance of observed migration timing from green wave is the difference between observed and expected arrival day, a positive value denotes a later arrival than green wave, and vice versa; ^‡^This component did not exist for models including only one-year data. ^§^For models including only one-year data, this column denotes the variance of individual birds. ^‖^results not applicable because the Correlation method evaluated by Stochastic Migrations is designed for validating green wave surfers/weak surfers identified by Simple Conventional Correlations; ^¶^Simulations cannot be performed due to lack of required data.

Supplementary Table 6**. Results of Simple Conventional Correlation, Correlation method evaluated by Stochastic Migrations, and Metric Selection approach based on Stochastic Migrations, based on different buffer size of remote sensing data extraction at stopover sites.**

| Species | Population | Feeding guild | Simple Conventional Correlation | | | | Correlation method evaluated by Stochastic Migrations | | | | Metric Selection approach based on Stochastic Migrations^*^ | | | |
| --- | --- | --- | --- | --- | --- | --- | --- | --- | --- | --- | --- | --- | --- | --- |
|  |  |  | 5 km | 10 km | 15 km | 30 km | 5 km | 10 km | 15 km | 30 km | 5 km | 10 km | 15 km | 30 km |
| Barnacle Goose | Greenland | Grazer | ○ | ○ | ◖ | ◖ | ○ | ○ | ● | ● | ○ | ○ | ○ | ○ |
| Barnacle Goose | Svalbard | Grazer | ◖ | ◖ | ○ | ○ | ● | ● | ● | ○ | ● | ● | ● | ● |
| Barnacle Goose | Barents Sea | Grazer | ◖ | ◖ | ◖ | ◖ | ● | ● | ● | ● | ● | ● | ● | ● |
| Greater White-fronted Goose | Barents Sea | Grazer | ◖ | ◖ | ◖ | ◖ | ● | ● | ● | ● | ● | ● | ● | ● |
| Greater White-fronted Goose | East Asia | Grazer | ○ | ○ | ○ | ○ | ○ | ○ | ○ | ○ | ○ | ○ | ○ | ○ |
| Whooper Swan | East Asia | Facultative herbivore | ● | ● | ◖ | ◖ | ● | ● | ● | ● | ○ | ○ | ○ | ○ |
| Tundra Swan | East Asia | Facultative herbivore | ○ | ○ | ○ | ○ | ○ | ○ | ○ | ○ | ○ | ○ | ○ | ○ |
| Swan Goose | East Asia | Facultative herbivore | ○ | ○ | ○ | ○ | ○ | ○ | ○ | ○ | ○ | ○ | ○ | ○ |
| Taiga Bean Goose | Scandinavia | Facultative herbivore | ○ | ○ | ○ | ○ | ○ | ○ | ○ | ○ | ○ | ○ | ○ | ○ |
| Tundra Bean Goose | East Asia | Facultative herbivore | ○ | ○ | ○ | ○ | ○ | ○ | ○ | ○ | ○ | ○ | ○ | ○ |
| Pink-footed Goose | Svalbard | Facultative herbivore | ◖ | ◖ | ◖ | ○ | ● | ● | ● | ● | ○ | ○ | ○ | ○ |
| Greater White-fronted Goose | Greenland | Facultative herbivore | ○ | ○ | ○ | ○ | ○ | ○ | ○ | ○ | ○ | ○ | ○ | ○ |
| Mallard | East Asia | Omnivore | ○ | ○ | ○ | ○ | ○ | ○ | ○ | ○ | ○ | ○ | ○ | ○ |
| Northern Pintail | North America | Omnivore | ◖ | ◖ | ○ | ◖ | ○ | ○ | ○ | ○ | ○ | ○ | ○ | ○ |
| Criteria to evaluate the level of support | |  |  |  |  |  |  |  |  |  |  |  |  |  |
| Differences among feeding guilds | |  | 🗶 | 🗶 | 🗶 | 🗶 | 🗶 | 🗶 | 🗶 | 🗶 | ✓ | ✓ | ✓ | ✓ |
| Effect of bill morphology | | Scheme 1^*^ | 🗶 | 🗶 | Not applicable^†^ | | 🗶 | 🗶 | **?** | 🗶 | ✓ | ✓ | ✓ | ✓ |
|  |  | Scheme 2^*^ | 🗶 | 🗶 |  |  | Not applicable^‡^ | | | | Not applicable^‡^ | | | |
|  |  | Scheme 3^*^ | 🗶 | 🗶 | 🗶 | 🗶 |  |  |  |  |  |  |  |  |

Level of support (from high to low) is marked as ● for a surfer, ◖ for a weak surfer, and ○ for a non-surfer. ✓/🗶 denotes that the results met/failed to meet the evaluation criteria; ? denotes that the results weakly met the evaluation criteria cannot be determined because ΔAICc of the best model <2, although the bill morphology was included in the best model.^*^See Supplementary Table 4 for explanations for the three modelling schemes. ^†^The modelling schemes are not applicable because of the lack of green wave surfers. ^‡^The modelling schemes are not applicable because of the lack of weak surfers.

Supplementary Table 7**. Biological family and upper mandible depth-length ratio of study species.**

| Species | Biological family | Upper mandible depth-length ratio ± SD (N=5) |
| --- | --- | --- |
| Whooper Swan | Swan | 0.402 ± 0.049 |
| Tundra Swan | Swan | 0.449 ± 0.028 |
| Barnacle Goose | Goose | 0.598 ± 0.032 |
| Swan Goose | Goose | 0.468 ± 0.040 |
| Taiga Bean Goose | Goose | 0.464 ± 0.022 |
| Tundra Bean Goose | Goose | 0.465 ± 0.032 |
| Pink-footed Goose | Goose | 0.520 ± 0.046 |
| Greater White-fronted Goose (grazing) | Goose | 0.564 ± 0.044 |
| Greenland Greater White-fronted Goose (facultative herbivore) | Goose | 0.506 ± 0.031 |
| Mallard | Duck | 0.413 ± 0.012 |
| Northern Pintail | Duck | 0.388 ± 0.072 |


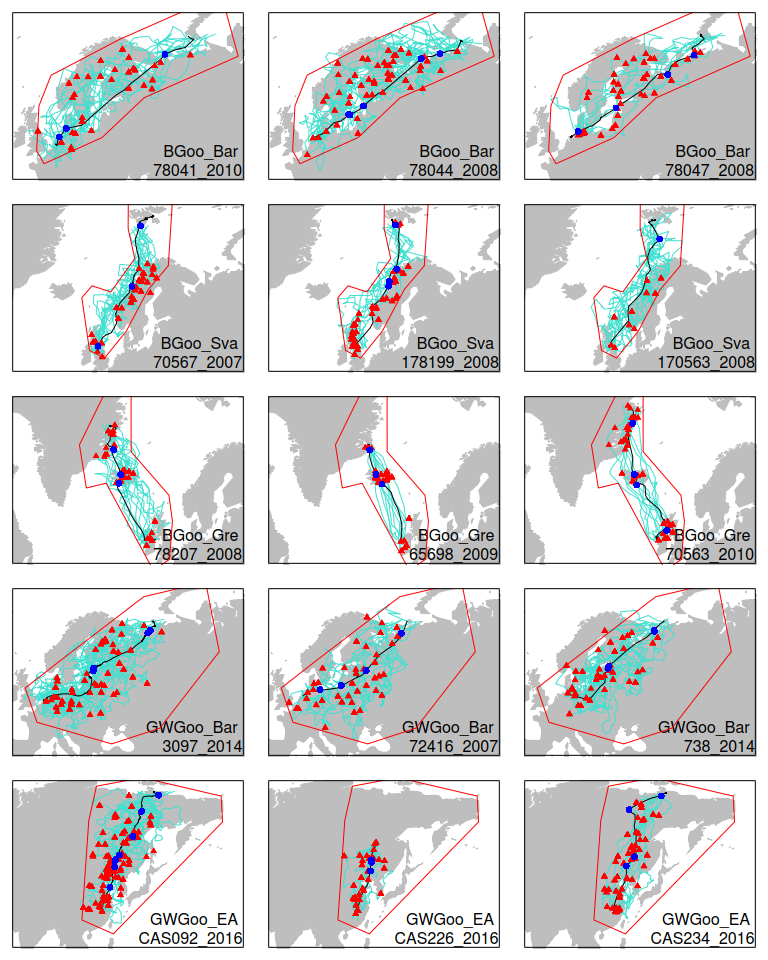


Supplementary Figure 1**. Examples of stochastic migrations and stopover sites generated for five populations.** The stochastically generated stopover sites are used in stochastic stopover site modelling and stochastic timing and stopover site modelling. Each panel shows the observed migration track (black line), the observed stopover sites (blue points) and 10 stochastic tracks (turquoise lines) with their associated stopover sites (red triangles). The red polygons are the estimated migration range of the population (Supplementary Fig. 9). See Supplementary Table 3 for population abbreviations.


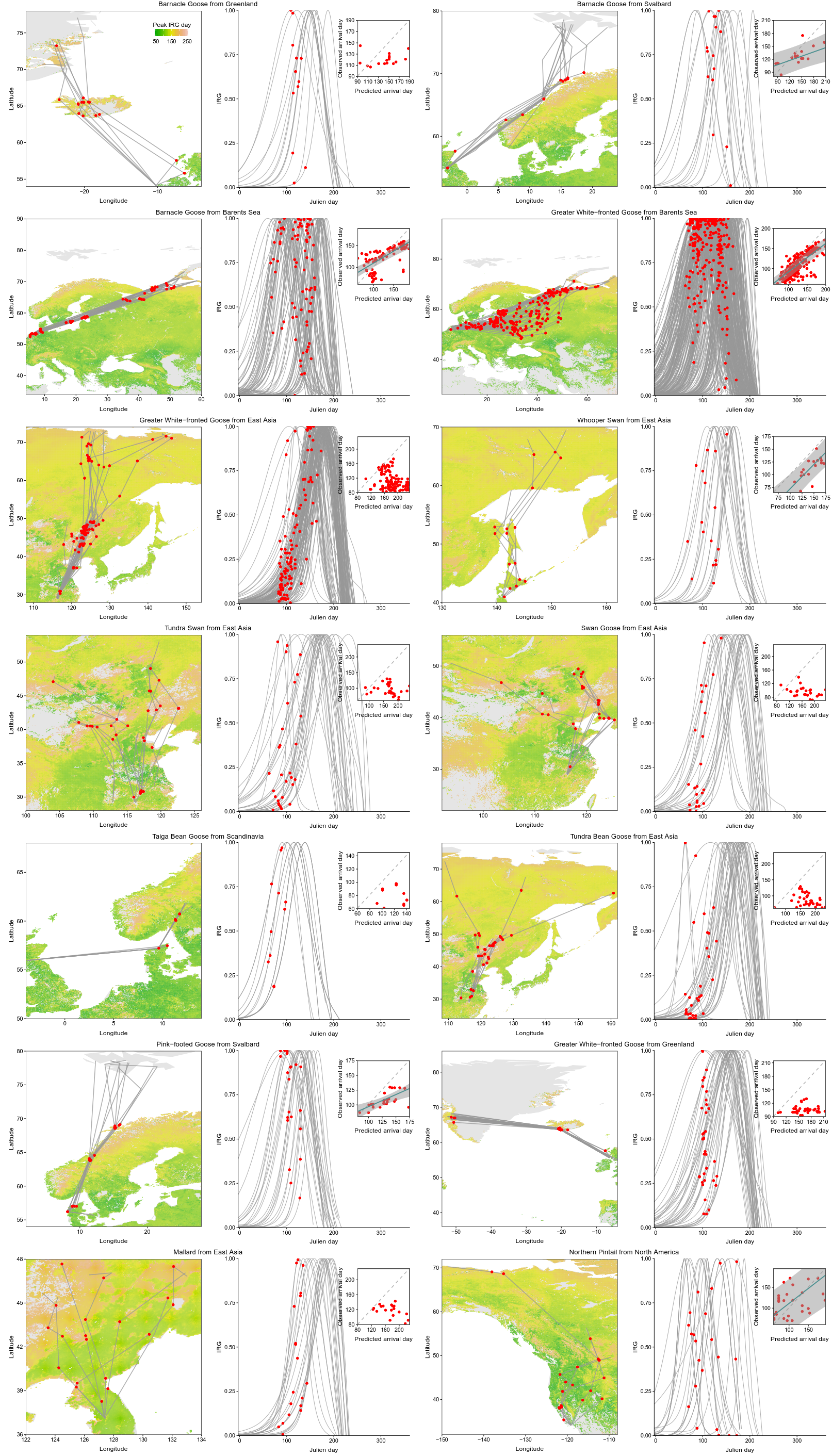


Supplementary Figure 2**. Migration, spatial green-wave pattern *en route*, and migration-green wave correlations of each Anatidae population.** For each species, left panel: stopover sites (red points) of individual migration episodes (grey lines) are shown on the map of peak IRG day, i.e. the expected arrival day according to the green wave (see Supplementary Fig. 11 for maps of other green wave metrics); right panel: the time-IRG curve (grey lines, for details see Supplementary Fig. 7) at each stopover sites, observed arrival days and the corresponding IRG values (red points); the migration-green wave correlation, slope (if significant) with confidence bands of each population are shown in insets.


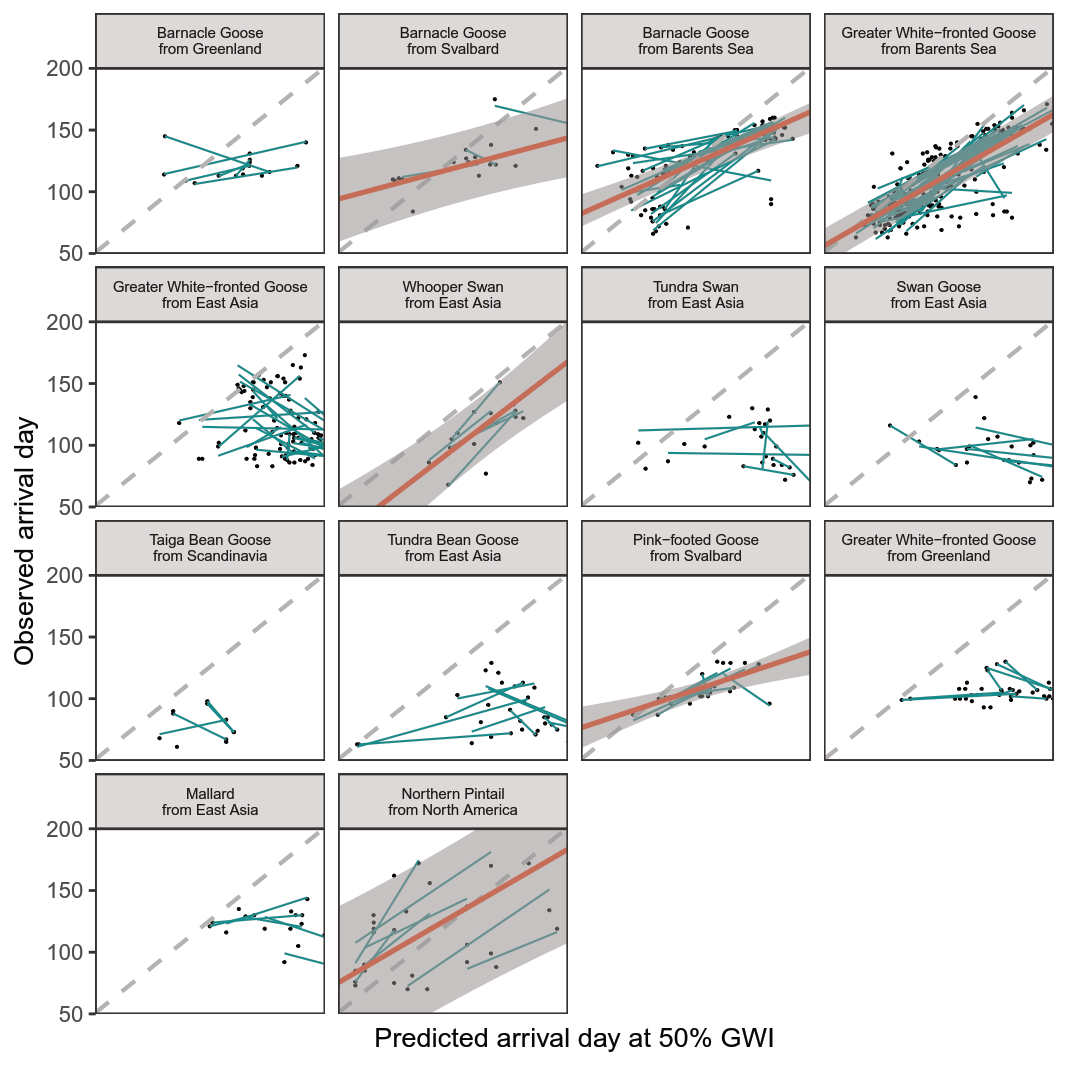


Supplementary Figure 3**. Migration–green wave correlations of each Anatidae population in population and individual levels.** The grey pecked lines with 1-slope and 0-intercept indicate the exact match of migration and green wave. Red lines show the significant positive slope of green wave surfers/weak surfers in population level; the grey bands are the prediction intervals of the models in population level. Blue lines are individual-level migration–green wave correlations.


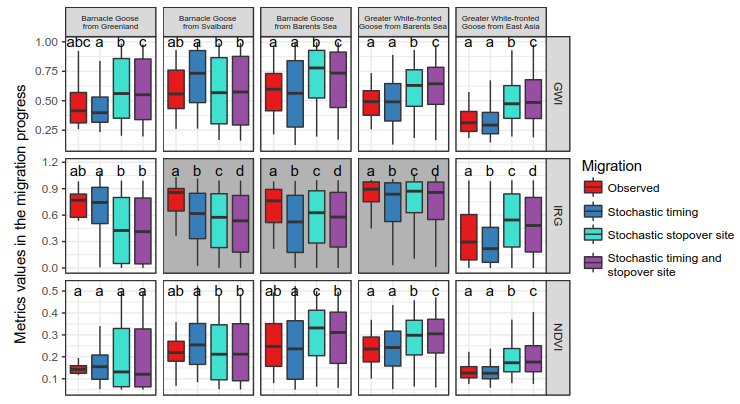


Supplementary Figure 4**. Green wave metric values in the migration progress for observed, stochastic timing, stochastic stopover site, and stochastic timing and stopover site migrations for grazers.** The metrics include GWI – green wave index, IRG – instantaneous rate of green-up (the first derivative of NDVI time series rescaled from 0 to 1) and NDVI. Lower case letters indicate significantly different groups using Kruskal-Wallis test followed by Dunn’s test of multiple comparisons. For populations without available migration tracks, only stochastic timing simulations were performed, compared and plotted. Grey panels indicate significant difference in metric values between observed and any stochastic migrations. Boxplots show median, first and third quartiles with whiskers reaching to the last data point within 1.5× interquartile range. For clear presentation, outliers out of 10% and 90% quantiles were excluded from the plots but kept in all analyses. Source data are provided as a Source Data file.


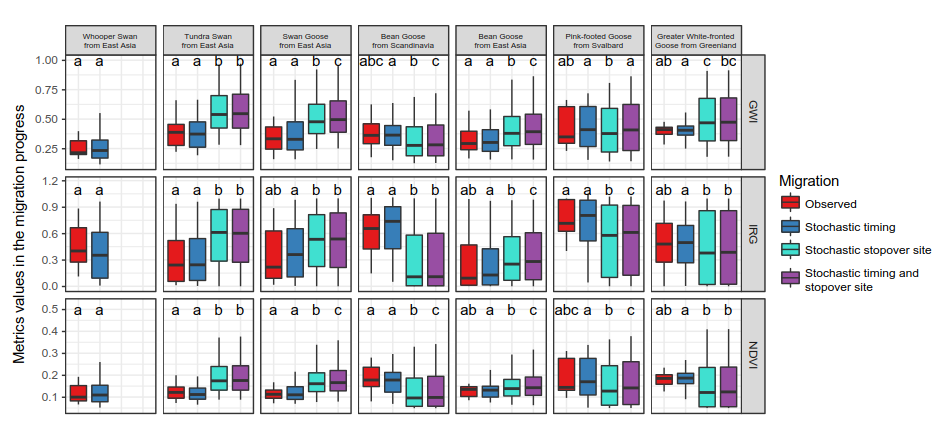


Supplementary Figure 5**. Green wave metric values in the migration progress for observed, stochastic timing, stochastic stopover site, and stochastic timing and stopover site migrations for facultative herbivores.** See Supplementary Fig. 4 for definitions and details of panels, symbols, colours, acronyms and boxplots. Source data are provided as a Source Data file.


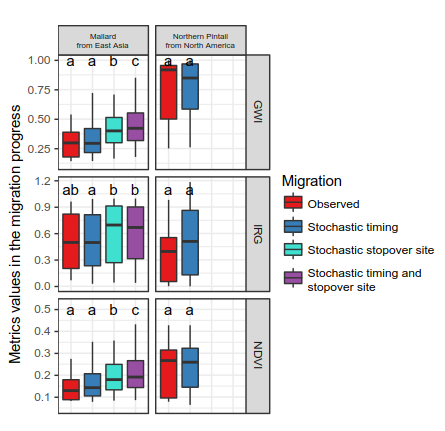


Supplementary Figure 6**. Green wave metric values in the migration progress for observed, stochastic timing, stochastic stopover site, and stochastic timing and stopover site migrations for omnivores.** See Supplementary Fig. 4 for definitions and details of panels, symbols, colours, acronyms and boxplots. Source data are provided as a Source Data file.


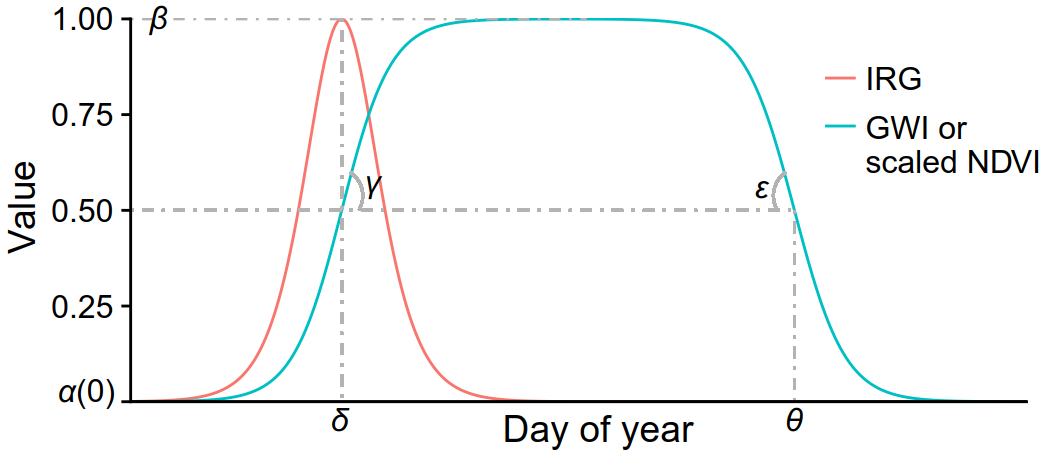


Supplementary Figure 7**. Example of the double-logistic model (Equation [1]) used to fit the yearly time series (blue line) of scaled NDVI, or green wave index (GWI), and the corresponding curve (red line) of the spring instantaneous rate of green-up (IRG), the derivative of the NDVI double-logistic curve.** *α* and *β* reflect the minimum and maximum NDVI values; *γ* and *ε* are the rates of increase/decrease of the curve at the inflection points; *δ* and *θ* are time of maximum/minimum green-up rate of Equation (1).

**
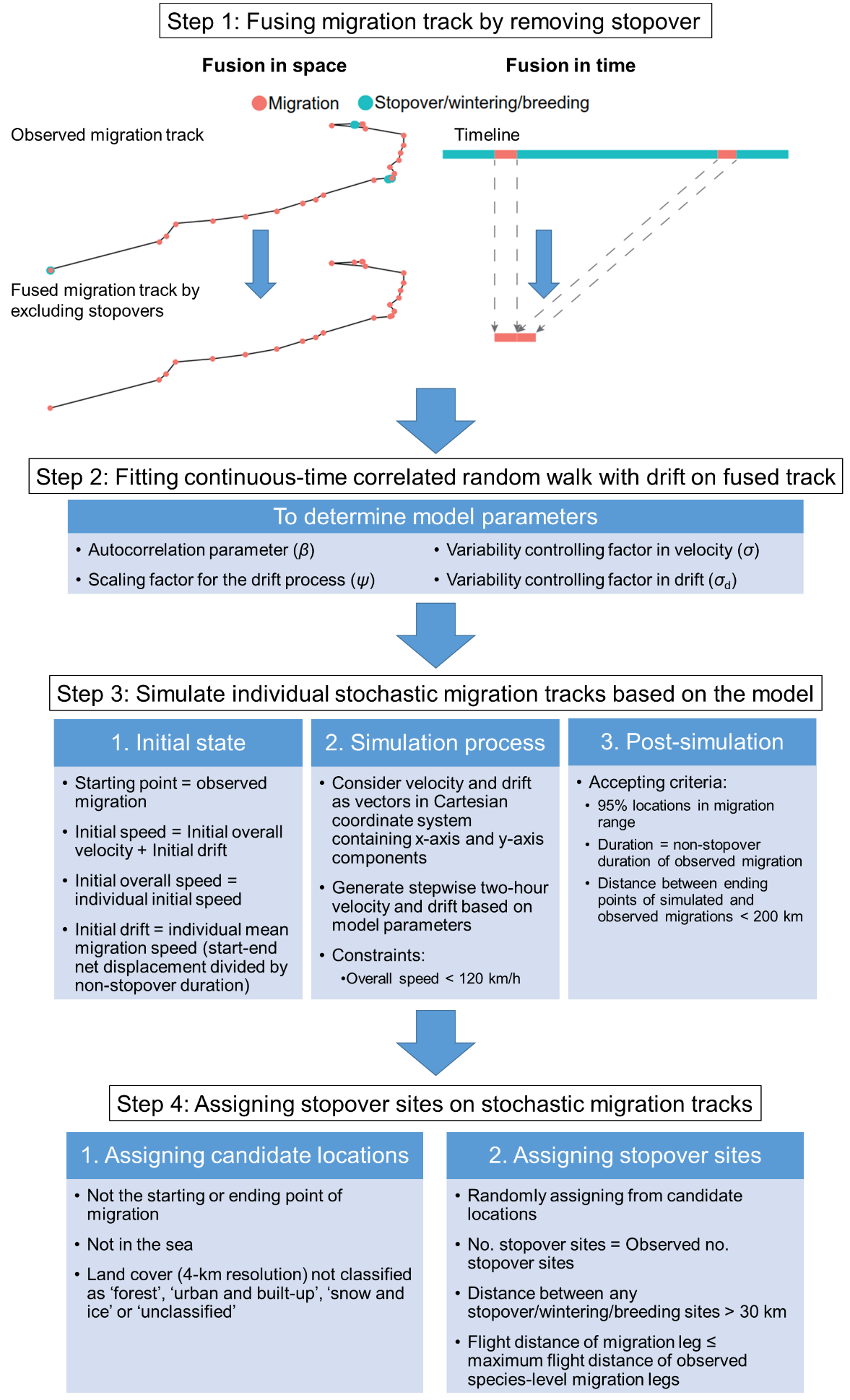
**

Supplementary Figure 8**. A brief description of the four-step process to generate stochastic migration tracks and stopover locations. See *Methods* for details.**


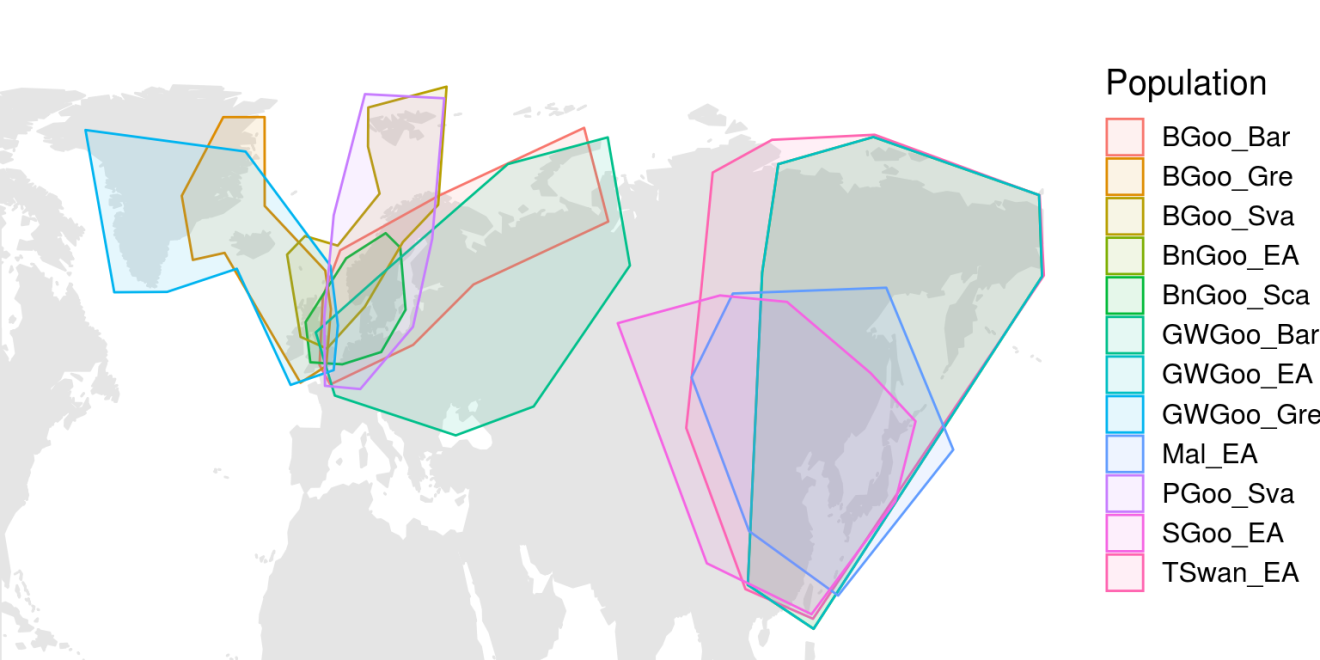


Supplementary Figure 9**. Estimated migration range of migratory waterbirds.** See *Methods* for constraining stochastic migration range, and Supplementary Table 3 for population abbreviations.


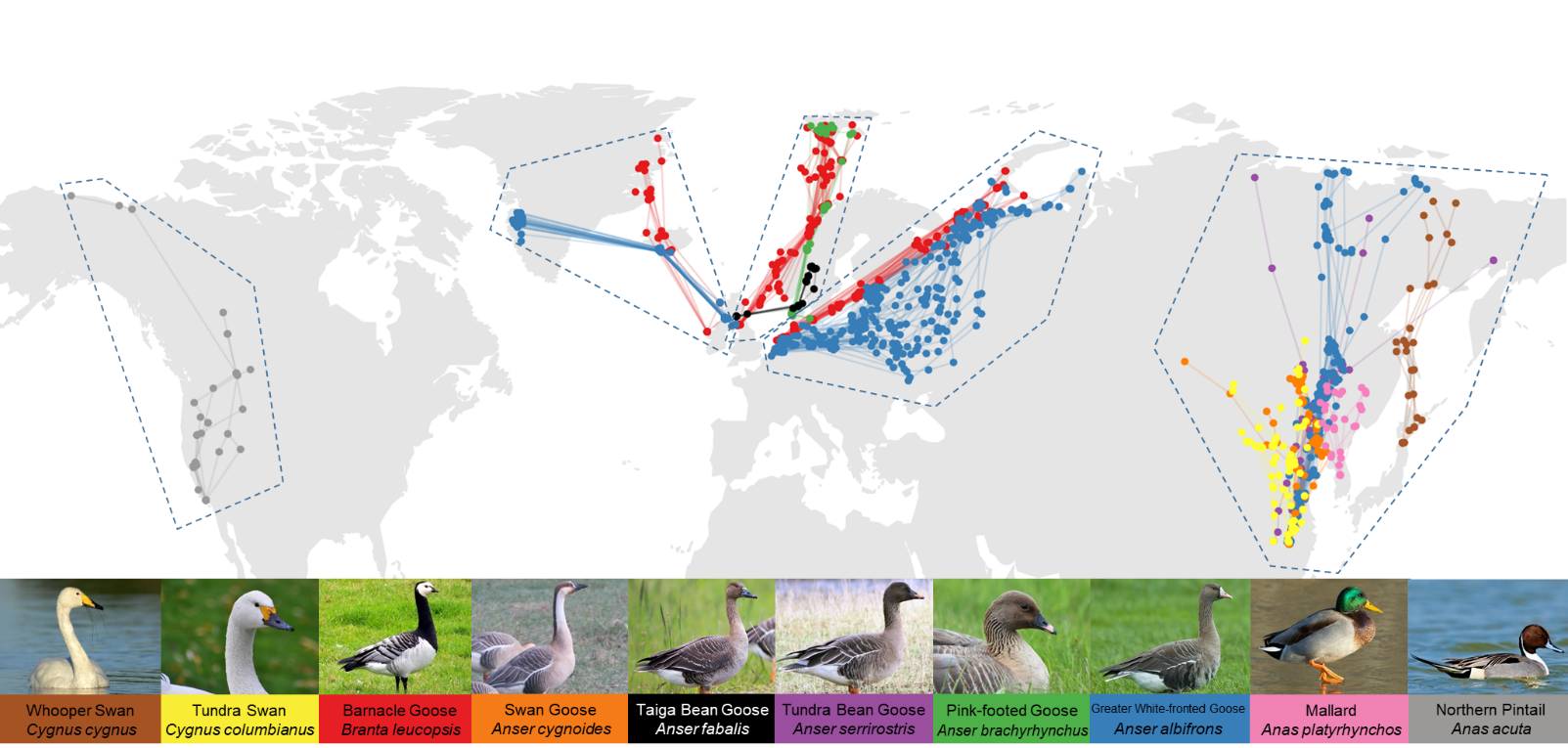


Supplementary Figure 10**. Geographical regions of migration of birds divided by blue polygons, based on their flyway and aggregation of stopover sites, including North America, Greenland, Svalbard, Barents Sea and East Asia, from left to right.**


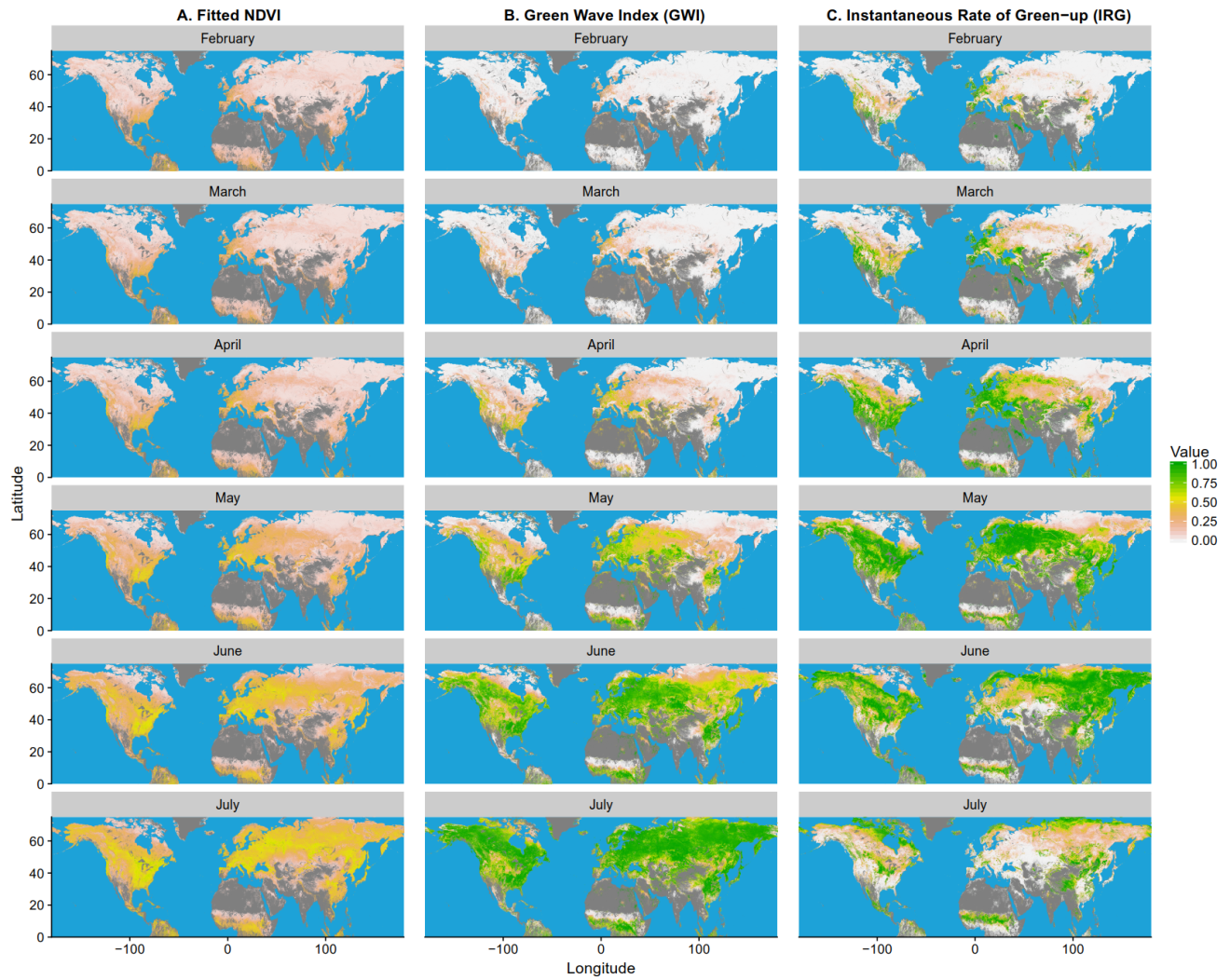


Supplementary Figure 11**. The monthly pattern of green wave metrics, (A) fitted NDVI, (B) Green Wave Index (GWI) and (C) Instantaneous Rate of Green-up (IRG), in Northern Hemisphere from 1^st^ February to 1^st^ July, 2016.** All values are calculated by pixel-based double-logistic models. Grey pixels are non-vegetated area.

# Supplementary References

1 Cabot, D. Data from: Forecasting spring from afar? Timing of migration and predictability of phenology along different migration routes of an avian herbivore [Greenland data]. *Movebank data repository* (2014).

2 Kolzsch, A. *et al.* Forecasting spring from afar? Timing of migration and predictability of phenology along different migration routes of an avian herbivore. *J Anim Ecol* **84**, 272-283 (2015).

3 van der Graaf, S. A. J., Stahl, J., Klimkowska, A., Bakker, J. P. & Drent, R. H. Surfing on a green wave - how plant growth drives spring migration in the Barnacle Goose Branta leucopsis. *Ardea* **94**, 567-577 (2006).

4 Griffin, L. Data from: Forecasting spring from afar? Timing of migration and predictability of phenology along different migration routes of an avian herbivore [Svalbard data]. *Movebank data repository* (2014).

5 Van Der Jeugd, H. P., Oosterbeek, K., Ens, B. J., Shamoun-Baranes, J. & Exo, K. Data from: Forecasting spring from afar? Timing of migration and predictability of phenology along different migration routes of an avian herbivore [Barents Sea data]. *Movebank data repository* (2014).

6 Kölzsch, A. *et al.* Towards a new understanding of migration timing: slower spring than autumn migration in geese reflects different decision rules for stopover use and departure. *Oikos* **125**, 1496-1507 (2016).

7 Kear, J. *Ducks, geese and swans*. (Oxford University Press, 2005).

8 Wang, X., Zhang, Y., Zhao, M., Cao, L. & Fox, A. D. The benefits of being big: effects of body size on energy budgets of three wintering goose species grazing Carex beds in the Yangtze River floodplain, China. *Journal of Ornithology* **154**, 1095-1103 (2013).

9 Kanai, Y. *et al.* The migration routes and important rest sites of whooper swans satellite-tracked from northern Japan. *Strix* **15**, 1-13 (1997).

10 del Hoyo, J., Elliott, A., Sargatal, J., Christie, D. A. & de Juana, E. *Handbook of the Birds of the World Alive.*, (Lynx Edicions, 2019).

11 Cong, P. *et al.* A comparison of the behaviour and habitat use by Bewick’s Swans *Cygnus columbianus bewickii* at wintering sites in China and Europe: preliminary observations. *Wildfowl* **61**, 52-73 (2011).

12 Zhang, Y. *et al.* Changing distribution and abundance of Swan Goose *Anser cygnoides* in the Yangtze River floodplain: the likely loss of a very important wintering site. *Bird Conserv Int* **21**, 36-48 (2011).

13 Marjakangas, A. *et al.* International single species action plan for the conservation of the Taiga Bean Goose *Anser fabalis fabalis*. (Bonn, Germany, 2016).

14 Zhao, M., Cao, L. & Fox, A. D. Distribution and diet of wintering Tundra Bean Geese *Anser fabalis serrirostris* at Shengjin Lake, Yangtze River floodplain, China. *Wildfowl* **60**, 52-63 (2010).

15 Chudzińska, M. E., Nabe-Nielsen, J., Nolet, B. A. & Madsen, J. Foraging behaviour and fuel accumulation of capital breeders during spring migration as derived from a combination of satellite- and ground-based observations. *J Avian Biol* **47**, 563-574 (2016).

16 Chudzińska, M. E. & Madsen, J. Data from: Foraging behaviour and fuel accumulation of capital breeders during spring migration as derived from a combination of satellite- and ground-based observations. *Movebank data repository* (2016).

17 Fox, A. D. *et al.* Effects of agricultural change on abundance, fitness components and distribution of two arctic-nesting goose populations. *Glob Change Biol* **11**, 881-893 (2005).

18 Dou, S., Cao, L., Cheng, Y. & Fox, A. D. Functional use of Shengjin Hu National Nature Reserve, China, by three species of dabbling ducks – preliminary observations. *Wildfowl* **60**, 124-135 (2010).

19 Miller, M. R., Takekawa, J. Y., Battaglia, D. S., Golightly, R. T. & Perry, W. M. Spring migration and summer destinations of Northern Pintails from the coast of southern California. *Southwest Nat* **55**, 501-509 (2010).

20 Johnson, P. C. D. Extension of Nakagawa & Schielzeth's R2GLMM to random slopes models. *Methods Ecol Evol* **5**, 944-946 (2014).

21 Nakagawa, S. & Schielzeth, H. A general and simple method for obtaining R2 from generalized linear mixed-effects models. *Methods Ecol Evol* **4**, 133-142 (2013).
