# Supplementary material for: Stochastic simulations reveal few green wave surfing populations among spring migrating herbivorous waterfowl
Source: Nat Commun. 2019 May 16;10:2187. doi: 10.1038/s41467-019-09971-8 (PMC6522631; doi:10.1038/s41467-019-09971-8)
Supplement: Supplementary file 3 — Description of Additional Supplementary Files [file 41467_2019_9971_MOESM3_ESM.pdf]

Supplementary Data 1. Details of unpublished tracking data of spring migration, logger information and capture information. [*]The total sum of movement distance during the entire tracked period, including movement during migration and stopover sites. [†]GPS/GPS+Argos, and hereafter, means that the study used both GPS loggers and GPS+Argos loggers, which provided both GPS locations and Argos locations. However, we only included GPS locations in all analyses in this study and in this overview report table, because (1) Argos locations involved much higher error than GPS locations, (2) the GPS+Argos loggers provided much fewer Argos locations than GPS locations, and (3) the simultaneous working of the two location systems will result in duplicated timestamped locations;

Supplementary Data 2. Continuous-time correlated random walk model parameters of individual migration tracks. σ is the variability controlling factor in velocity; σ_d is the variable controlling factor in drift; β is the autocorrelation parameter; ψ is the scaling factor for the drift process;
